# The Effects of the Shape and Size of the Clinical Target Volume on the Planning Target Volume Margin

**DOI:** 10.1371/journal.pone.0109244

**Published:** 2014-10-02

**Authors:** Buhong Zheng, Zhiyu Huang, Jinluan Li

**Affiliations:** Department of Radiation Oncology, Teaching Hospital of Fujian Medical University, Fujian Provincial Cancer Hospital, Fuzhou, Fujian, P. R. China; University of Nebraska Medical Center, United States of America

## Abstract

**Purpose:**

To investigate the impact of clinical target volume (CTV) shape and size on CTV to planning target volume (PTV) margin expansion.

**Methods and Materials:**

Using numerical integration methods, margins accounting for random errors and systematic errors were calculated for CTVs of different shapes and sizes. We use 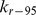 and 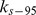 to represent the coefficients, for random errors and systematic errors, respectively, that ensure that every point of the CTV receives ≥95% of the prescribed dose.

**Results:**

The part of the margin accounting for random errors depends on CTV shape and size; generally, a convex part of a CTV would have a larger margin than a concave part. However, the part of the margin accounting for systematic errors is independent of CTV shape and size.

**Conclusions:**

CTV shape and size should be considered when generating a PTV. For a complex CTV, the margins of the various parts of the CTV are different and related to local forms.

## Introduction

In recent decades, new irradiation techniques, such as 3-dimensional conformal radiation therapy and intensity-modulated radiation therapy, have allowed the delivery of a higher dose of radiation to the target site while sparing adjacent normal tissues. However, these techniques have also aroused some concern, as they are more sensitive to geometric uncertainties due to sharper dose gradients around the target volume. Margin recipes have been proposed by several groups [1]–[6], but discrepancies persist. Although the effects of the shape and size of the clinical target volume (CTV) have been mentioned by some authors as being relevant [3], [7], the influence of the CTV has never been widely considered and fully interpreted.

According to the International Commission on Radiation Units and Measurements (ICRU) reports [8], a margin should be added to the CTV to generate the planning target volume (PTV), in order to account for all geometric uncertainties and ensure that the CTV receives a sufficient dose. The total errors are the deviations between the intended position and actual position of the CTV, and these are primarily caused by patient localization (setup) errors and organ motion [2], [9]–[11]. For each patient, the systematic error persists throughout the treatment, while the random errors occur stochastically in each fraction. Two parameters may be obtained from both the systematic errors and the random errors: the mean and the standard deviation (SD). The mean of the systematic errors (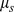) is probably not zero, but almost all authors assume it to be zero when deducing the margin recipe [1]–[4]. Assuming that the systematic errors and random errors are normally distributed with 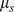 = 0 and 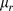 = 0, the margin recipe are approximately written as:
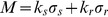
(1)where 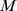 represents the margin; 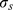 represents the SD of the systematic errors; 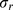 represents the SD of the random errors; and 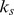 and 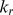 represent the coefficients of 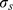 and 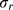, respectively.

In this paper, we will first assume that the systematic errors are zero, and derive the PTV margins for the random errors. Then, we will assume that the random errors are zero, and derive the PTV margins for the systematic errors.

## Methods and Materials

### 1.1. Presumptions and formulae

Before deriving the PTV margin, we first made a number of assumptions:

1. The distributions of the systematic errors and the random errors are independent, three-dimensional normal distributions.
2. The CTVs are rigid bodies, and only translational errors are considered (rotations or deformations are not considered).
3. The displacements of the bodies and CTVs do not affect the dose distributions. In other words, the displacements of the bodies and CTVs only move the dose distributions translationally inside the bodies, but do not distort them. This assumption can be approximately satisfied when a multiple beam irradiation technique is used and the CTVs are not adjacent to the body surfaces.
4. Patients are treated with numerous fractions.

Based on the above assumptions, the dose for a certain point of a CTV may be calculated by:
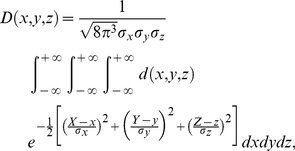
(2)where 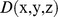 is the dose received by a point at position 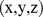; 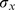, 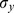 and 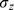 are the SDs of the errors (systematic or random errors) in the x, y and z directions, respectively; and 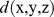 is the dose distribution function. For simplicity, we will assume that the isodose surface obtained after planning will be ideally identical to the PTV surface; in other words, the dose inside the PTV is 100%, and the dose outside the PTV is 0%. Thus, 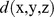 becomes a step function:
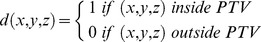
(3)


Since it is sometimes difficult or even impossible to find the antiderivative of an integrand, we approximate the integral by numerical integration methods. Since the probability of a point being outside the ellipsoid with semi-axes 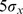, 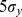 and 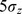 is less than 0.002%, we did not calculate the dose beyond this region. Therefore, the numerical integration form for equation (2) would be:
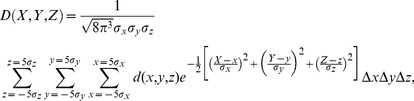
(4)where 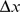, 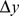 and 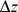 are subinterval widths in the x, y and z directions, respectively. In order to obtain sufficient accuracy for clinical use, we generally set 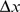, 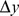 and 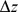 to 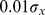, 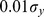 and 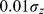, respectively.

### 1.2. Margins accounting for random errors

We first derive the PTV margins to account for random errors, assuming that the systematic errors are zero. In this paper, we use the notation 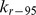 to represent the coefficient that can ensure that every point in the CTV receives at least 95% of the prescribed dose. The 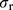 parameters in the x, y and z directions are represented by 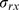, 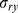 and 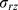, respectively.

#### 1.2.1. Point CTV

**Figure 1:**
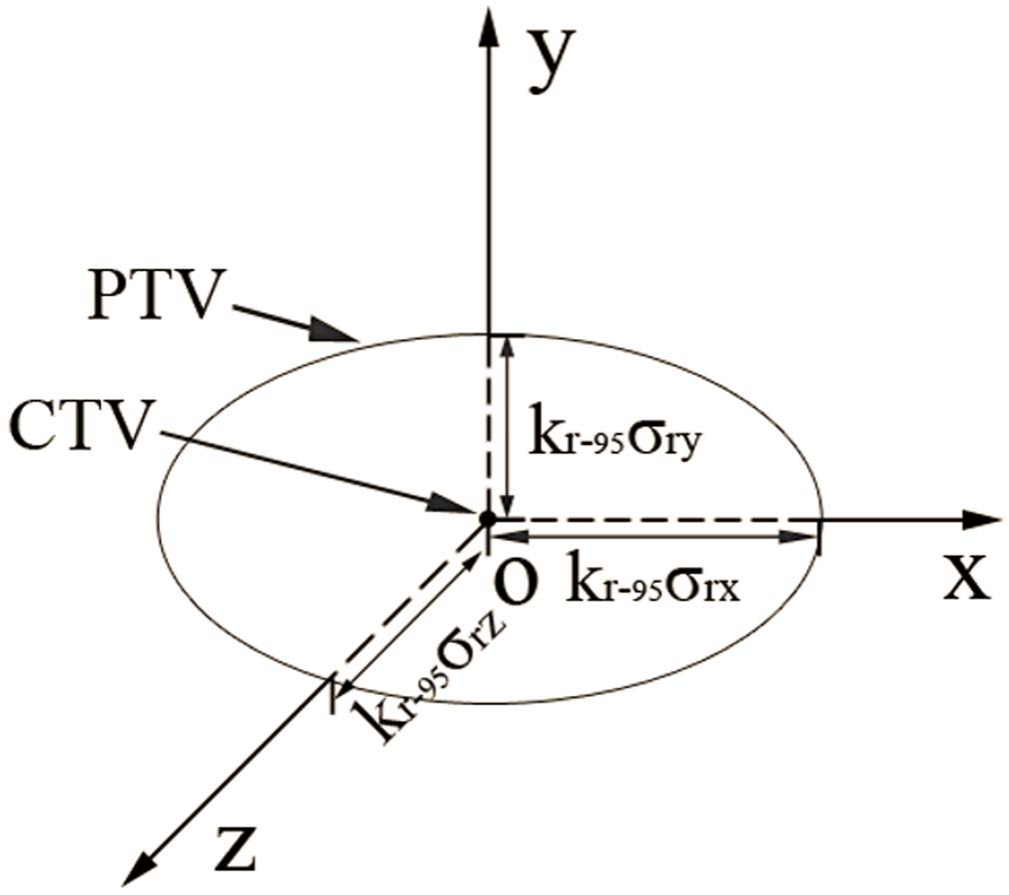
The expansion of a single point CTV.

When the CTV is just a single point, we can simply expand this point with an ellipsoid (Figure 1) of semi-axes 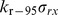, 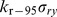 and 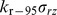 to ensure that this point has a 95% probability of being inside the ellipsoid (for an ideal dose distribution). The 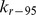 for a single point CTV is 2.79, which may be easily calculated by equation (4).

#### 1.2.2. The method for expanding CTVs of other shapes and sizes

It is straightforward to generate the PTV for a point CTV. However, the CTVs that are handled in clinical practice are usually lumps of differing shapes and sizes. Taking the entire lump into consideration would be a complicated approach. We think it is feasible to start from a point on the surface of the CTV. We can expand a surface point with an ellipsoid to ensure that this point receives 95% of the prescribed dose. After expanding every point on the surface with an ellipsoid, we can add up all these ellipsoids and the CTV to generate a PTV. For any CTV that is closed and does not contain a hole (most CTVs in clinical practice meet this criterion), if the surface points can receive 95% of the prescribed dose, then the dose received by the interior points should be ≥95% of the prescribed dose. Thus, for any CTV, we only need to consider the points on the surface. However, the size of the ellipsoidal margin of a certain surface point will be influenced by the expansions of other nearby surface points, as shown below.

#### 1.2.3. Spherical CTV

**Figure 2:**
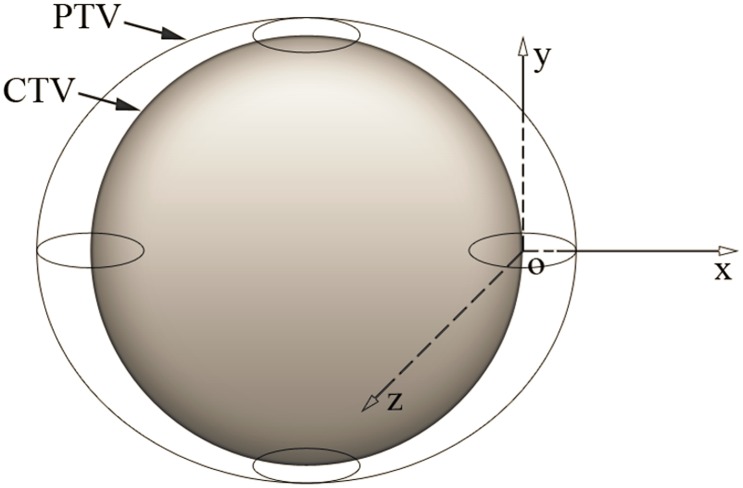
The expansion of a spherical CTV.

CTVs similar to a sphere may be the most common type encountered in clinical practice. For a spherical CTV, due to the “supplement” of the CTV itself and the ellipsoidal margins of other nearby points, it would not be necessary to expand every surface point with such a large ellipsoid as a point CTV. Suppose that the radius of the spherical CTV is 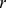; since the surface of the spherical CTV is symmetric in any direction, we can imagine that the PTV we generate is similar to an ellipsoid (Figure 2) with semi-axes 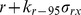, 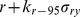 and 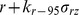.

#### Linear CTV

First, we consider a fictitious linear CTV 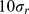 in length, and assume that 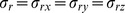. Since the surface of this line is just the line itself, we expand every point of the line with a spherical margin. If we maintain the concept that the 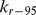 values for all points are equal, the PTV we generate would look like a capsule. However, when we calculate the 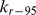 values for the midpoint and endpoint separately, they are 2.45 and 2.66. Therefore, the tailored PTV of a linear CTV is not a capsule, but resembles a dumbbell in some respects. The margin of the endpoint is a sphere of radius 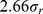, while that of the midpoint is a sphere of radius 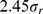, and there are transitions between them.

#### 1.2.4. CTVs of other shapes

Using numerical integration methods, we can also calculate the 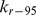 values for cylindrical CTVs. As with a linear CTV, the 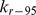 values for different surface points of a cylindrical CTV may be different. To avoid being influenced by nearby surface points, the 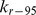 values we calculate are for points on the middle of the lateral faces of the cylindrical CTVs that are more than 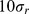 high. Sometimes, we encounter CTVs with concave regions. Assuming 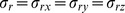, we can also calculate 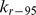 values for spherical and cylindrical concave regions of CTVs with different radii. For simplicity, the 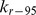 values for spherical concave regions are calculated based on concentric sphere CTVs, and the 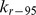 values for cylindrical concave regions are calculated based on CTVs with cylindrical holes that are more than 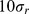 high.

### 1.3. Margins accounting for systematic errors

**Figure 3:**
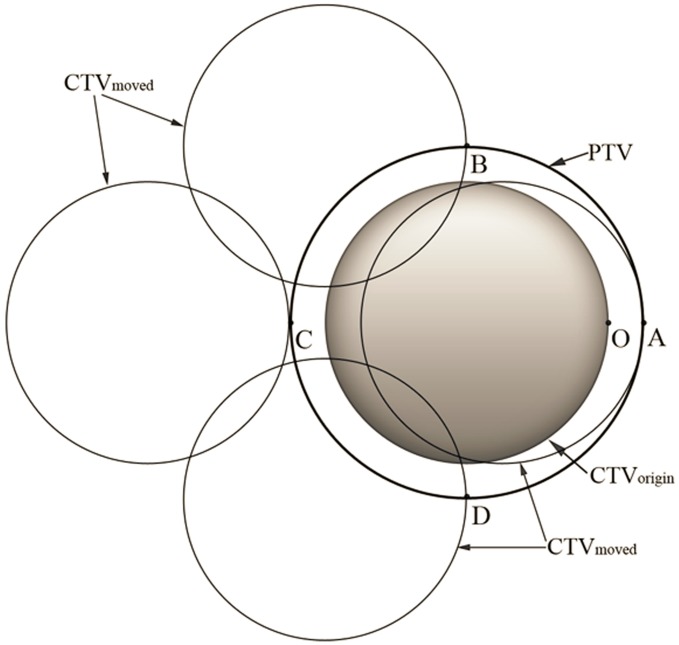
The method for calculating the margins for random errors adds up the probability that point O is at any position of the PTV to obtain the probability that point O is inside the PTV. When point O is at position B, C, or D, part (position B and D) or even all (position C) of the CTV is outside the PTV.

**Figure 4:**
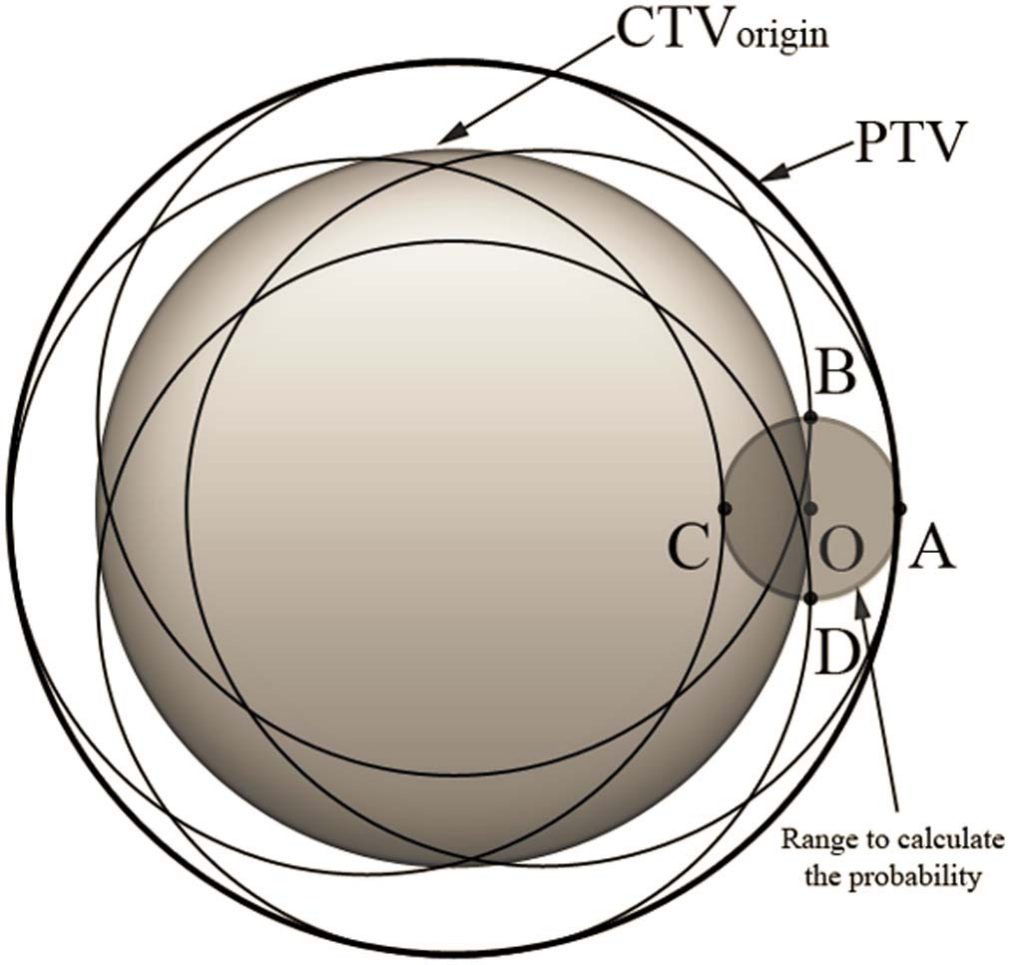
To ensure that “95% of the systematic displacements of the CTVs for a population of patients are completely inside the PTVs”, we should only add up the probability that point O is at any position inside the small sphere. When point O is at any position (A, B, C, or D etc.) inside this small sphere, the CTV is completely inside the PTV.

As mentioned above, the population of systematic errors consists of the systematic displacements of the CTV positions in all patients. Taking into consideration the proposal of Van Herk *et al.* [1], we define the 95% confidence level as: “95% of the systematic displacements of the CTVs for a population of patients are inside the PTVs”. We use 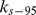 to denote the coefficient multiplying to the 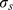 to achieve this goal. An important question is whether the method we use for random errors is still applicable for systematic errors. The previous method is suitable when applied to random errors and when patients are treated with sufficient fractions, because if we treat a patient with a large number of fractions, the dose distribution will be blurred and every part of the CTV will receive at least 95% of the prescribed dose. However, for systematic errors, such “blurring” will not occur. The criterion that “95% of the systematic displacements of the CTVs for a population of patients are inside the PTVs” should be regarded as “95% of the systematic displacements of the CTVs for a population of patients are *completely* inside the PTVs”. This means that we need to ensure that, simultaneously, the probability of every point on the surface of the CTV being inside the PTV is 95%. We call this prerequisite “synchronization”. Take a spherical CTV, for example. As shown in Figure 3, without “synchronization”, point O can be at any position inside the PTV when we calculate the 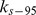 of point O. However, when point O is at positions B, C, or D, part of the CTV (position B and D) or even all of the CTV (position C) is outside the PTV. With the constraint of “synchronization”, the probability of point O being at positions such as B, C, or D should not be counted. If we expand every point on the surface of this CTV with a spherical margin of radius 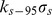 (assume that 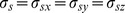, where 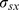, 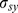 and 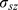 are 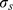 in the x, y and z directions, respectively), under the constraint that the whole CTV must be inside the PTV, the volume for calculating the probability is none other than the spherical margin of a point CTV (Figure 4). In fact, after “synchronization”, the volume for calculating the probability for CTVs of any shape or size can be reduced to a single point.

## Results

### 1. Margins accounting for random errors

#### 1.1 Spherical and cylindrical CTVs

**Figure 5:**
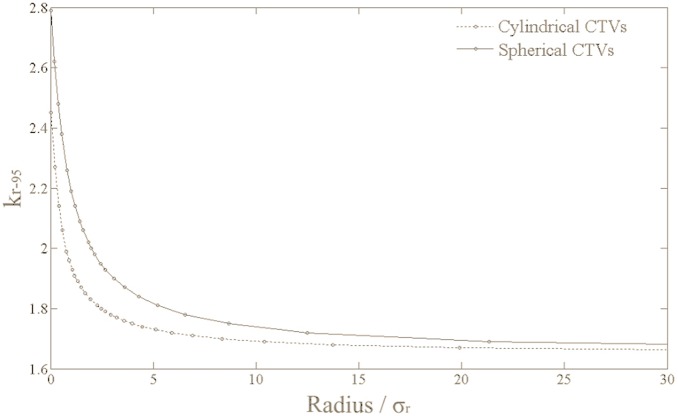
The values for spherical and cylindrical CTVs of different radii.

Assuming 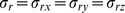, we calculated the 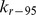 values for spherical and cylindrical CTVs with different radii, and plotted the relation between the 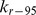 and the radius in Figure 5. It may be seen that the 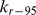 declines from a value of 2.79 for a point CTV to approximately 1.65 for large spherical CTVs. The curve for cylindrical CTVs resembles that for spherical CTVs, except that the 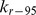 for a cylindrical CTV of radius 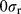 (i.e., a linear CTV) is 2.45.

#### 1.2 Concave regions of CTVs

**Figure 6:**
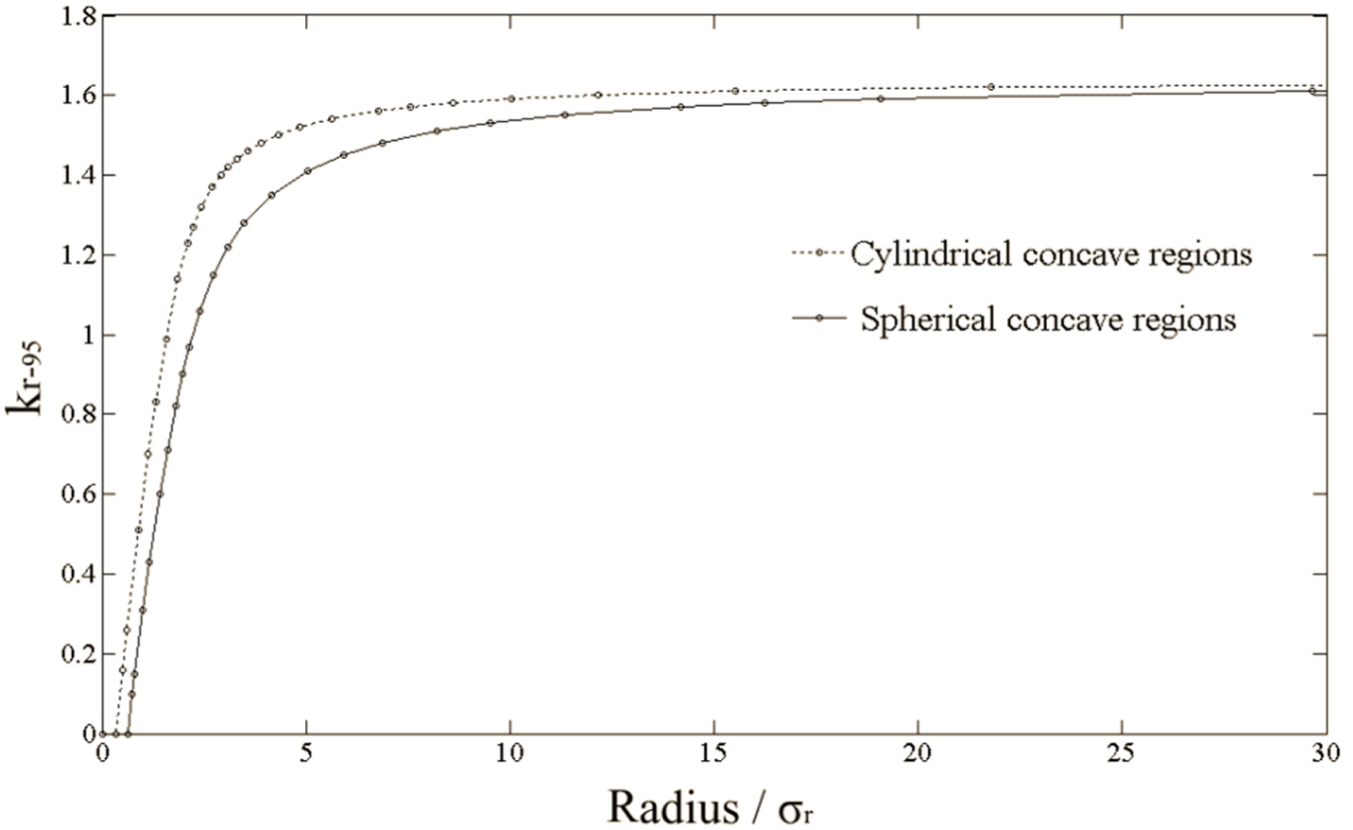
The values for spherical and cylindrical concave regions of CTVs.

Assuming 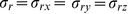, we also calculated the 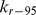 values for spherical and cylindrical concave regions of CTVs (Figure 4b, 4c) with different radii. Figure 6 shows the relation between the 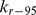 values and the radii. It may be seen that for these two particular concave regions, the 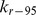 values are less than 1.65. Nevertheless, the CTVs we encounter in clinical practice are not likely to have spherical or cylindrical holes in them. The most common situation is that a CTV has one or more concave part(s) accompanied by one or more convex part(s). The 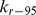 values for the surface points of the convex part(s) are larger than 1.65, but for the concave part(s) they may be less than 1.65 (the actual value depends on the local shape), with transitions between them.

### 2. Systematic errors

In order to ensure that every part of the CTV is simultaneously inside the PTV, the 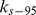 is always 2.79, irrespective of the shape and size of the CTV.

## Discussion

### Margins accounting for random errors

In section 2.2.1, we assigned the same coefficient 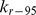 to 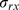, 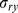 and 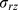 to generate the margin for a point CTV. We would call this “uniform expansion” because the probability densities for points on the surface of the margin are equal. However, this is not the only approach. Under certain circumstances, we can assign different coefficients to 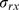, 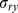 and 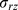 to achieve the same goal. For example, we can expand a point CTV with an ellipsoid of semi-axes 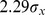, 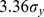 and 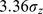 to ensure that the CTV can receive the same 95% prescribed dose. We would call this kind of expansion “non-uniform expansion” in that it is no longer statistically uniform in all directions. In the same way, we can perform “non-uniform expansion” for CTVs of other shapes and sizes. However, we should be aware that “non-uniform expansion” would result in a larger PTV (the proof of this statement is beyond the scope of this paper). Therefore, “non-uniform expansion” should only be used in particular circumstances (for example, if the spinal cord is highly adjacent to the CTV in one direction).

Austin-Seymour *et al.* [4] described a model to generate the PTV by cylindrical expansion of the CTV. These authors suggested that in order to attain a nominal probability of 95%, the height of the cylindrical expansion should be 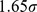 and the radius should be 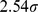. However, it should be noted that cylindrical expansion is one type of “non-uniform expansion” that would result in an unnecessarily large PTV. Craig *et al.* [12] have also discussed “uniform margins” and “non-uniform margins”. However, their definitions were quite different from ours. The “uniform margins” mentioned by these authors refer to the addition of equal margins in the x, y and z directions, no matter whether 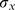, 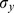 and 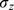 are equal or not. In addition, the “non-uniform margins” they defined involved adding 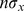, 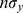 and 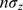 in the x, y and z directions, respectively, which equates to what we would term “uniform expansion”. In other words, their use of “uniform” was in the context of “geometrically uniform”, whereas ours is in the context of “statistically uniform”. Antolak *et al.* [3], [13] argued that to ensure every point on the surface of the CTV is within the PTV approximately 95% of the time, the CTV should be expanded with a sphere of normalized radius 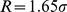. Applying our method, we know that 1.65 is the 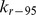 for a huge spherical CTV. To our knowledge, the study of Antolak *et al.* is one of the few that have taken into consideration the shapes and sizes of the CTVs. Using numerical integration methods, these authors deduced that if a spherical CTV of radius 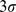 is expanded with a 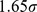 margin, the probability of any CTV surface point being within the PTV is about 92%. Although Antonlak *et al.* were of the opinion that this small difference (92% *vs.* 95%) could be ignored, their results are nonetheless consistent with ours.

We derived the 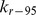 based on the presumption that the isodose surface is ideally identical to the PTV surface, i.e., 100% inside the PTV and 0% outside the PTV. However, in practice, the dose gradient around the PTV would not be so steep, and obviously this would reduce the value of 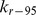.

Theoretically, our method can be applied to CTVs of any shape and size; however, it is burdensome and sometimes almost impossible to calculate 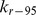 values for all surface points of a bulky and complex CTV. It is hoped that our methods could be incorporated into the Treatment Planning System to take into account both the shape and size of the CTV and the real dose gradient to automatically generate an appropriate PTV.

### Margins accounting for systematic errors

Van Herk *et al.* [1] suggested a margin recipe of 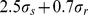 to meet the criterion that “for 90% of the patient population, the minimum dose to the CTV must be 95% of the nominal dose or higher”. This recipe also took only translational errors into account. With respect to systematic errors, their recipe is consistent with ours, because if we set our confidence level more generously to 90%, we can obtain the same 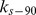 of 2.5.

So far, we have only discussed how to generate the PTV margins to account for systematic errors when the random errors are zero. However, if we want to generate a margin to account for both systematic errors and random errors so that “95% of the systematic displacements of the CTVs for a population of patients are inside the PTVs” and “every point of these 95% CTVs receive at least 95% of the prescribed dose”, the first step required is to expand every point on the surface of the CTV with an ellipsoid of semi-axes 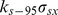, 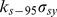 and 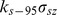, which need not pay attention to the random errors. Thus, 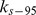 is independent of random errors and will always be 2.79.

## Conclusion

The margin for a CTV surface point accounting for random errors depends on the local shape and size. For a complex CTV, different margins should be tailored to different parts. The derivation of the margin to account for systematic errors should be under the restriction that the total CTV must be simultaneously inside the PTV. This restriction endows the margins for systematic errors, independent of CTV size, CTV shape, and the number of treatment fractions.
